# From knowledge to impact: revolutionizing nursing practices in biomedical waste management for sustainable healthcare excellence

**DOI:** 10.1186/s12912-025-03073-1

**Published:** 2025-04-29

**Authors:** Alaa Eldin Moustafa Hamed, Linda Sefouhi, Mohammed Ibrahim Touni Ibrahim, Ahmed Shaaban Attia, Abeer Moustafa Barakat, Essam Eltantawy Elsayed

**Affiliations:** 1 Lecturer Psychiatric and Mental Health Nursing, Faculty of Nursing, Helwan University, Cairo, Egypt; 2 LRNAT, Institute of Industrial Hygiene and Safety, University of Batna 2, Batna, Algeria; 3 Faculty of Nursing, Jerash University, Jerash, Jordan; 4 Minia University Hospitals, Minia University, Minia, Egypt; 5 Lecturer of Critical Care Nursing, Faculty of Nursing, Helwan University, Cairo, Egypt; 6 Lecturer of Maternal and Newborn Health Nursing, Faculty of Nursing, Helwan University, Cairo, Egypt; 7 College of Nursing, Hafr Albatin University, Hafr Albatin, Saudi Arabia

**Keywords:** Biomedical waste management, Nursing competence, Educational intervention, Healthcare environment, Waste management practices

## Abstract

**Background:**

Effective biomedical waste management is essential for minimizing environmental contamination and safeguarding public health. Nurses play a pivotal role in this process, yet their competence often requires enhancement, particularly in resource-limited settings.

**Aim:**

This study evaluates the effectiveness of an educational program in improving nursing competence in biomedical waste management at El-Minia University Hospitals.

**Methods:**

A quasi-experimental design was employed with pre- and post-intervention assessments measuring nurses’ knowledge and practices. The intervention, structured into three sessions, combined lectures, demonstrations, and hands-on practice. Seventy-five nurses participated, and data were collected using validated tools. Statistical analyses included paired t-tests and correlation assessments.

**Results:**

Post-intervention assessments revealed a significant improvement in nurses’ knowledge (*p* < 0.001), attitudes, and practices (*p* < 0.001) related to biomedical waste management. Additionally, the correlation between knowledge and practice increased post-intervention (*r* = 0.62, *p* < 0.001), highlighting a stronger positive association between these variables.

**Conclusion:**

Structured educational programs effectively enhance nursing competence in biomedical waste management, contributing to safer healthcare environments. Further research should explore long-term sustainability and curriculum integration.

**Clinical trial number:**

NCT06718660 on 5/12/2024.

## Background

Healthcare institutions are essential for treatment but are also major contributors to infectious waste. The global expansion of healthcare facilities has increased the use of disposable medical equipment, leading to a surge in biomedical waste production. Additionally, advancements in medical technology have further escalated the volume of waste generated per case worldwide [1].

Biomedical waste, which includes sharps, human tissues, and other infectious materials, presents serious hazards, including the transmission of diseases such as HIV and hepatitis. Improper disposal can lead to environmental contamination, affecting water sources and ecosystems, while also endangering individuals handling the waste [2]. This makes effective biomedical waste management (BMW) critical for public health and environmental sustainability.

Despite the recognized importance of proper biomedical waste management, many healthcare facilities, particularly in developing countries, face challenges due to inadequate training, insufficient awareness, and poor waste segregation practices [3]. Studies indicate that a lack of structured educational interventions significantly contributes to these deficiencies [4]. However, there remains a gap in understanding the extent to which targeted educational programs can enhance nursing competence in biomedical waste management, particularly within university hospital settings [5].

Environmental contamination from biomedical waste has significant social, economic, and health consequences. Polluted water and soil negatively impact agriculture and fishing industries, leading to job losses and increased poverty within affected communities. Additionally, biomedical waste exposure can cause anxiety and stress, reducing quality of life and trust in institutions [6]. Among the key challenges in biomedical waste management are ineffective waste systems, lack of awareness, insufficient financial and human resources, and poor disposal practices [7]. Proper biomedical waste management through segregation, collection, treatment, and disposal is essential for protecting public health and the environment. Techniques such as incineration, autoclaving, and secure landfill disposal help mitigate the risks of disease transmission and contamination [8]. Infection control nurses play a vital role as educators, advocates, and enforcers of safe waste practices. Their responsibilities include training healthcare workers, promoting effective policies, and ensuring compliance with waste management protocols to prevent infections and environmental harm [9–12].

This study is guided by Kolb’s Experiential Learning Theory (ELT), which emphasizes hands-on learning as a key driver of competency development [13]. Given that biomedical waste management requires both theoretical knowledge and practical application, ELT provides a suitable framework for evaluating how experiential training can improve nurses’ competence. While previous research has focused on general waste management policies and challenges, few studies have assessed the impact of structured educational interventions on nursing competence in biomedical waste management within Egyptian healthcare settings. Thus, this study addresses a critical research gap by evaluating the effectiveness of an educational program in improving nurses’ knowledge, and practices regarding biomedical waste management at El-Minia University Hospitals. The findings will contribute to evidence-based strategies for integrating waste management training into nursing curricula and hospital policies, ultimately enhancing patient safety and environmental protection.

## Methods

### Aim of the study

This study aimed to evaluate the effectiveness of an educational program designed to enhance nursing competence in biomedical waste management at El-Minia University Hospitals.

### Research hypothesis

The educational intervention program will significantly enhance nursing competence in biomedical waste management at El-Minia University Hospital.

### Setting and study design

The study was conducted in El-Minia University Hospital affiliated to El-Minia University in El-Minia Governance, Egypt. A quasi-Experimental design was utilized to carry out the study.

### Participants

**Fig. 1:**
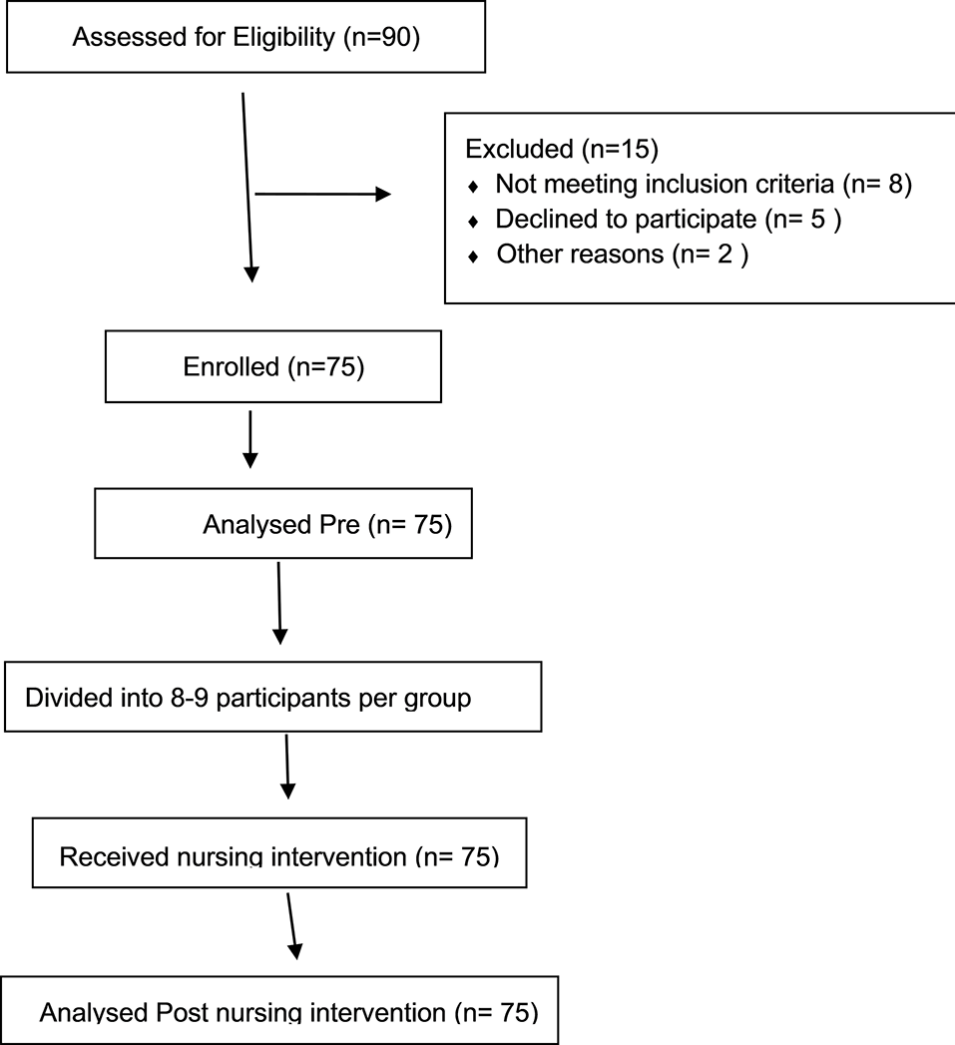
Participants flow

Epi Info software version 7 was used to calculate the sample size, assuming a potential 30% improvement in knowledge. The calculation was based on data from a pilot study and set at a 95% confidence interval (CI) with a power level of 80% (β = 0.20) to detect a statistically significant difference. A significance level of *p* ≤ 0.05 was applied. Considering an expected 10% dropout rate, the required sample size was determined to be 75 participants. Figure 1 illustrates the participant flow throughout the study, detailing the number of nurses recruited, those who completed the intervention and any attrition that occurred Fig. 1.

### Study instruments

This study utilized a well-structured face-to-face interview questionnaire in the participants’ native language, Arabic. The instruments were translated from English to Arabic by the Center for Specialized Languages at the Faculty of Arts, Helwan University. A panel of experts in psychology, psychiatry, and psychiatric nursing evaluated the instruments to ensure their validity and appropriateness for use with nursing staff. The experts also refined the questions to ensure cultural relevance for Egyptian nurses. A pilot study involving 8 participants was conducted to confirm the reliability and clarity of the interview questionnaire. Following this, the researchers conducted face-to-face interviews with nursing staff across various hospital departments.

This research instrument encompasses three primary sections, the first one was socio-demographic characteristics of participant nurses, containing the participant’s basic and educational information, including age, gender, level of education, years of experience, educational level, department where they work and previous training in the field of waste management. The second section was Nurses’ Knowledge Regarding Biomedical Waste and Its Management questionnaire, which was developed by Khalil and colleagues, (2024) [10]. This tool aimed to evaluate nurses’ knowledge of biomedical waste and its management. It comprised 29 multiple-choice questions, with 21 questions having only one correct answer and 8 questions allowing multiple correct responses. The six covered areas included: knowledge of waste management policies, types of waste, hazards associated with improper waste disposal, methods of waste management, steps involved in waste handling, and locations for biomedical waste collection. Additionally, it assessed nurses’ sources of information. As for the scoring system it was as follows: two points for a fully correct answer, one point for a partially correct answer, and zero points for an incorrect or incomplete answer. Questions with only one correct answer were scored as one point for a correct response and zero for an incorrect response. The total score, ranging from 0 to 58, was converted into percentages. Nurses’ knowledge levels were categorized as Low level: <70% of the total score (< 41 points), Moderate level: 70-80% of the total score (41–46 points) while High level: >80% of the total score (> 46 points). Cronbach’s Alpha test was used to determine the tool’s reliability; the result was (0.83).

The third part of the study instrument nurses’ self-reported practices regarding biomedical waste management scale, developed by Hassan and colleagues, (2020) in the light of CDC guidelines [14]. It comprised 64 items that assessed various practices, including the following six parts: universal precautions when dealing with biomedical waste (10 items), adhering to color-coded segregation (9 items), following instructions on waste containers (11 items), proper disposal of sharp wastes (14 items), handling and disposing of liquid medical waste (11 items), and managing spills (9 items). The scoring system categorized responses as “always done” (2 points), “sometimes done” (1 point), or “never done” (0 points), resulting in a total score ranging from 0 to 128. These scores were converted into percentages, with practices classified as competent (≥ 85% of the total score, ≥ 108 points) or incompetent (< 85%, < 108 points). The tool exhibited acceptable reliability, as indicated by a Cronbach’s alpha of 0.776, reflecting satisfactory internal consistency.

### Procedures

The data collection for this study was instituted after obtaining ethical approval from faculty of nursing Helwan university followed by the approval from the hospital authorities to conduct the current study after an explanation of the aim of the study. For this study, a well-structured interview using a previously prepared questionnaire with the participants using the participants’ mother tongue (Arabic) was employed. The researchers used the questionnaire, incorporating relevant Nurses’ Knowledge Regarding Biomedical Waste and Its Management questionnaire, and the self-reported practices of nursing staff regarding biomedical waste management scale from related studies. The instruments were translated from English to Arabic at the Center for Specialized Languages, Faculty of Arts, Helwan University. A panel of experts then evaluated the instrument’s validity and suitability for use with nurses. Then the questionnaire was distributed by the researchers through performing interviews with the participants in El Minia University Hospital.

The survey was structured into two distinct parts, with the first part serving as the informed consent. This section clarified the study’s objectives, assured participants of confidentiality, and informed them of their right to withdraw from the study at any point without any concern regarding potential risks. Consequently, the informed consent was integrated directly into the same document as the research tool. Participants were required to acknowledge and agree to these terms prior to proceeding with the questionnaire, ensuring their comprehensive understanding before beginning.

The educational intervention aimed at enhancing nursing competence in biomedical waste management was carefully structured and implemented in four successive phases at El-Minia University Hospital. In the Preparatory Phase, the research team conducted an extensive review of national and international guidelines, current studies, and resources on biomedical waste management. In this study, performance refers to the nurses’ competency in biomedical waste management, specifically their knowledge and self-reported practices. Knowledge was assessed using the Nurses’ Knowledge Regarding Biomedical Waste and Its Management Scale, while practices were evaluated via a self-reported practice questionnaire adapted from CDC guidelines. Performance improvement was determined by the statistical comparison of pre- and post-intervention scores. This phase included examining evidence-based research articles, textbooks, and online sources to design the study tools and ensure the research team was thoroughly familiar with the theoretical and practical aspects of the topic.

Following the preparatory work, the Assessment Phase which was conducted to evaluate the baseline knowledge and practices of the nursing staff at El-Minia University Hospital regarding biomedical waste management. This assessment revealed specific gaps in knowledge and practices, providing a foundation for the educational program’s tailored focus. In the Planning Phase, objectives, priorities, and desired outcomes were formulated to bridge the identified gaps in knowledge and practices. To achieve this educational materials, including clear and accessible booklets with illustrations, were developed specifically for the program. The study was conducted over a five-month period, starting at the beginning of August and concluding at the end of December, allowing sufficient time for participant recruitment, intervention delivery, and assessment of outcomes. The primary goals of the intervention were to empower nurses with the knowledge and skills to define biomedical waste, understand its various types and sources, recognize potential hazards, and implement proper handling and segregation techniques.

The Implementation Phase took place in a dedicated classroom-style setting within the hospital, arranged by the training department to support an interactive learning environment. To ensure effective learning, nurses were divided into 10 groups, each consisting of seven to eight participants. The grouping was based on work shifts and departmental assignments to minimize disruption to clinical duties while ensuring balanced participation. Each group attended a total of three structured sessions, delivered over a three-week period to allow time for practice and reinforcement of learning. The same group of nurses participated in all three sessions to ensure continuity and progressive skill development. Each session lasted 45–60 min and followed an interactive format, combining lectures, visual demonstrations, hands-on practice, and group discussions. Session 1 focused on defining biomedical waste, identifying types and sources, and understanding associated risks. Session 2 introduced waste handling technologies, color-coded segregation systems, and safe disposal methods. Session 3 highlighted critical infection control practices, such as proper hand hygiene and the use of personal protective equipment (PPE), including gloves and masks.

Finally, in the Evaluation Phase, approximately one month after the program’s completion, researchers reassessed the nurses’ knowledge and practices using the same tools from the initial assessment. This follow-up allowed for a comparison of pre- and post-intervention results, providing insight into the program’s effectiveness and measuring its impact on the nursing staff’s competence in biomedical waste management at El-Minia University Hospital. Through this structured approach, the nurses gained essential knowledge and practical skills, fostering safer and more effective biomedical waste management practices within the hospital setting.

### Ethical consideration

The Research Ethics Committee (REC) at the Faculty of Nursing, Helwan University, Egypt, approved the study protocol in its session number 42 on 14/7/2024. Prior to participation, all participants provided written informed consent as proof of their voluntary involvement in the study. Strict procedures were followed to ensure the protection of participants’ privacy, and all personally identifiable information was kept confidential, accessible only to the research team. Protecting participants’ confidentiality and privacy was of utmost importance. All study procedures were conducted in accordance with the ethical principles outlined in the Declaration of Helsinki and its subsequent amendments. Additionally, the study is registered at ClinicalTrials.gov under registration number NCT06718660, with the registration date of 5/12/2024.

### Statistical analysis

Data analysis was performed using SPSS 26.0 (IBM Inc., Chicago, IL, USA) to examine the survey responses from the recruited nurses at pre and post intervention. Descriptive statistics, including frequencies (percentages) and mean ± standard deviations (SD), were utilized to summarize both the value general characteristics of the participants and the scores obtained on various scales. Paired t-tests for comparing the mean scores between two periods within the same group. Correlation between different numerical variables was tested using Pearson product-moment correlation coefficient and spearman correlation for categorical variables. Probability (p-) less than 0.05 was considered significant and less than 0.001 considered as highly significant).

## Results

Table 1 displays the demographic characteristics of the nursing staff sample. The majority of nurses are aged between 30 and 39 years (40%), predominantly female (66.7%), and hold a Bachelor’s degree (60%). Regarding professional experience, most nurses have between 1 and 5 years of experience (40%), with fewer nurses represented in higher experience levels. In terms of department distribution, the largest proportion work in the General Ward (40%), followed by Emergency (26.7%), Surgery (20%), and Maternity (13.3%).

**Table 1:** socio-demographic characteristics of participant nurses (*N* = 75)

Demographic characteristics	No	%	Total knowledge		Total practice	
			Mean ± SD	*P*	Mean ± SD	*P*
**Age group (years)**						
20-<30	20	26.7	10.70 ± 5.18	0.615	37.75 ± 10.89	0.131
30-<40	30	40.0	9.20 ± 7.76		41.70 ± 9.92	
40-<50	15	20.0	9.47 ± 4.03		33.47 ± 11.32	
50–60	10	13.3	8.76 ± 4.22		38.50 ± 13.41	
**Gender**						
Male	25	33.3	9.32 ± 6.18	0.846	38.72 ± 12.28	0.937
Female	50	66.7	9.56 ± 4.35		38.50 ± 10.69	
**Level of education**						
Diploma	15	20.0	6.20 ± 2.17	0.015*	36.90 ± 12.21	0.039*
Bachelor of science in nursing (BSN)	45	60.0	8.98 ± 5.02		38.44 ± 10.93	
Master of science in nursing (MSN)	10	13.3	10.73 ± 4.89		39.13 ± 12.12	
Doctorate in nursing (PHD)	5	6.7	11.50 ± 5.26		41.40 ± 11.06	
**Years of experience**						
1-<5 years	30	40.0	11.57 ± 5.54	0.003*	37.53 ± 11.03	0.016*
5-<10 years	20	26.7	9.60 ± 3.23		38.15 ± 13.30	
10-<15 years	15	20.0	8.10 ± 5.08		41.90 ± 11.56	
> 15 years	10	13.3	6.07 ± 3.73		39.00 ± 8.37	
**Work area**						
General ward	30	40.0	9.03 ± 6.18	0.719	39.41 ± 9.79	0.293
Emergency	20	26.7	10.08 ± 4.30		37.56 ± 12.65	
Surgery	15	20.0	10.17 ± 3.38		41.92 ± 8.56	
Maternity	10	13.3	8.00 ± 4.99		31.67 ± 15.06	

*: Significant at *P* ≤ 0.05

Table 2 shows comparison between nurses’ total knowledge and total practices regarding biomedical waste management pre- and post-intervention. Concerning total knowledge, there was a highly statistically significant difference in total knowledge pre and post intervention which the total mean score of total knowledge in posttest (14.59 ± 4.38) about waste management were higher than mean scores of the pretest (9.48 ± 4.99) with (*P* < 0.001). Similarly, there was a highly statistically significant difference in total practice pre and post intervention, the mean scores of the total practice in posttest (38.57 ± 11.16) were higher than mean scores of the pretest (63.91 ± 22.38) at (*P* < 0.001).

**Table 2:** Comparison of nurses’ total knowledge and practices regarding biomedical waste management Pre- and Post-Intervention (N = 75)”

Items	Pretest	Posttest	t-test	*P*
Total knowledge Mean ± SD	9.48 ± 4.99	14.59 ± 4.38	6.250	< 0.001**
Total practice Mean ± SD	38.57 ± 11.16	63.91 ± 22.38	8.091	< 0.001**

*: Significant at *P* ≤ 0.05 **: Highly significant at *P* < 0.01

Table 3 illustrates the correlation between nurses’ total knowledge and practices regarding biomedical waste management pre- and post-intervention. Pre-intervention, a moderate positive correlation (*r* = 0.45) was found between knowledge and practices, which was statistically significant (*p* < 0.001). Post-intervention, this correlation strengthened to a higher positive value (*r* = 0.62), with a highly significant result (*p* < 0.001). These findings indicate that as nurses’ knowledge of biomedical waste management improves, their practices also significantly improve, especially following the intervention. The consistent positive correlation at both stages suggests that the intervention not only enhanced knowledge but also positively influenced the participants’ ability to translate this knowledge into practice.

**Table 3:** Correlation between nurses’ total knowledge and practices regarding biomedical waste management

Total practice		Total knowledge	
		Pre	Post
Pre	r	0.45	
	*P*-value	< 0.001**	
Post	r		0.62
	*P*-value		< 0.001**

*: Significant at *P* ≤ 0.05 **: Highly significant at *P* < 0.01

Table 4 presents the relationship between nurses’ total knowledge and practice regarding biomedical waste management and socio-demographic characteristics. A statistically significant association was found between both total knowledge and total practice with the level of education (knowledge: *p* = 0.030; practice: *p* = 0.015). However, no significant relationships were observed between either total knowledge or total practice with age, gender, years of experience, or work area, with p-values of 0.481, 0.809, 0.773, 0.250, and 0.558, 0.531, 0.630, and 0.148, respectively.

**Table 4:** Relationship between nurses’ total knowledge and practice regarding biomedical waste management and Socio-Demographic characteristics

Demographic characteristics	Total knowledge				*X2*	*P*-value	Total Practice				*X2*	*P*-value
	Unsatisfactory		Satisfactory				Incompetent		Competent			
	No	%	No	%			No	%	No	%		
**Age group**												
20–29	12	34.3	8	20	4.687	0.481	17	28.3	3	20	1.563	0.558
30–39	13	37.1	17	42.5			23	38.3	7	46.7		
40–49	4	11.5	11	27.5			13	21.7	2	13.3		
>50	6	17.1	4	10			7	11.7	3	20		
**Gender**												
Male	11	31.4	14	35	1.107	0.809	19	32.8	6	35.3	0.038	0.531
Female	24	68.6	26	65			39	67.2	11	64.7		
**Level of education**												
Technical institute of nursing	12	34.3	3	7.5	8.705	0.030*	13	22.4	2	11.8	10.345	0.015*
Bachelor of science in nursing (BSN)	21	60	24	60			36	62.1	9	52.9		
Master of science in nursing (MSN)	2	5.7	8	20			8	13.8	2	11.8		
Doctorate in nursing (PHD)	0	0	5	12.5			1	1.7	4	23.5		
**Years of experience**												
1–5 years	16	45.7	14	35	4.018	0.773	25	43.1	5	29.4	3.404	0.630
6–10 years	6	17.1	14	35			13	22.4	7	41.2		
11–15 years	9	25.7	6	15			13	22.4	2	11.8		
> 16 years	4	11.5	6	15			7	12.1	3	17.6		
**Work area**												
General Ward	12	34.3	20	50	5.397	0.250	28	43.3	6	40	0.781	0. 148
Emergency	12	34.3	13	32.5			20	33.3	5	33.3		
Surgery	9	25.7	3	7.5			10	16.7	2	13.3		
Maternity	2	5.7	4	10			4	6.7	2	13.3		

*: Significant at *P* ≤ 0.05

Table 5 presents the correlations between selected demographic characteristics and both pre- and post-intervention scores for total knowledge and total practice. Significant positive correlations were found between the level of education and both pre-intervention (*r* = 0.087, *P* < 0.05) and post-intervention (*r* = 0.072, *P* < 0.05) total knowledge scores. Additionally, significant correlations were observed between years of experience and both pre-intervention (*r* = 0.363, *P* < 0.01) and post-intervention (*r* = 0.540, *P* < 0.05) total practice scores.

**Table 5:** Correlations between demographic characteristics and Pre- and Post-Intervention scores for total knowledge and practices

Demographic characteristics	Total knowledge				Total practice			
	pre		post		pre		post	
	r1	p1	r2	p2	r1	p1	r2	p2
Age	0.080	0.496	0.078	0.507	0.061	0.601	0.049	0.675
Gender	0.061	0.602	0.011	0.928	0.045	0.701	0.037	0.751
Level of education	0.087	0.041*	0.072	0.02*	0.795	0.030*	0.904	0.014*
Years of experience	0.363	0.001*	0.082	0.484	0.111	0.045*	0.540	0.027*
Work Area	0.101	0.387	0.169	0.148	0.051	0.663	0.005	0.967

## Discussion

Improper management of medical waste generated by healthcare facilities poses significant risks to healthcare workers (HCWs), the general population, and the environment, as emphasized by global authorities such as the WHO and CDC [15]. Nurses play a critical role in ensuring the safe and regulated disposal of biomedical waste, making thorough knowledge and adherence to proper practices essential [11]. This highlights the importance of assessing nurses’ competence in biomedical waste management. Accordingly, our study aims to evaluate the effectiveness of an educational program designed to enhance nursing competence in biomedical waste management at El-Minia University Hospitals.

In relation to the nurses’ overall knowledge of biomedical waste management, the results of the current study revealed a considerable enhancement in their understanding after the implementation of the educational intervention. Initially, a significant proportion of nurses exhibited insufficient knowledge, while the rest demonstrated satisfactory understanding. However, following the intervention, there was a marked shift, with only a small percentage retaining inadequate knowledge, and the majority reaching satisfactory levels. The differences in knowledge levels pre and post the intervention were highly statistically significant.

Our study findings align with previous research in the field. For instance, Mahmoud and colleagues, (2022) conducted a study on the impact of an educational program for nurses in maternal and child healthcare centers regarding healthcare waste management, which showed a significant improvement in knowledge following the intervention [16]. Similarly, Hosny and colleagues, (2018) reported a notable increase in the mean score of knowledge related to waste management among healthcare workers after participating in an educational intervention, further reinforcing the effectiveness of such programs [17]. Additionally, Rajimol, (2022) assessed the influence of a planned teaching program on biomedical waste management knowledge among healthcare team members, with results indicating a substantial rise in knowledge levels post-intervention. This study highlights the importance of structured educational initiatives in enhancing awareness and understanding of critical practices among healthcare professionals [18].

These findings are further supported by Bhandari and colleagues, (2024), who investigated knowledge levels regarding biomedical waste management among nursing staff in a teaching hospital in Birgunj, Nepal [19]. Their study revealed that the majority of nurses already possessed an adequate level of knowledge, suggesting that the baseline knowledge of their study population may have been higher compared to the participants in the current study. This discrepancy could be attributed to differences in initial knowledge levels, educational backgrounds, or the effectiveness of prior training received by the nursing staff.

The findings of the current study highlight a significant improvement in nurses’ practices concerning biomedical waste management following the intervention. Prior to the intervention, only one-fifth of the nurses demonstrated competent practices, while the majority exhibited incompetent practices. However, post-intervention results revealed a marked improvement, with a substantial increase in the proportion of nurses displaying competent practices and a corresponding decrease in those categorized as incompetent. These changes were statistically significant, underscoring the efficacy of the intervention in enhancing nursing competence.

This notable improvement may be attributed to the structured training and awareness programs integrated into the intervention. These programs were meticulously designed to provide nurses with comprehensive knowledge and practical skills related to the safe handling and disposal of biomedical waste. The interactive and hands-on approach likely played a pivotal role in reinforcing learning and fostering confidence in implementing best practices.

This finding is consistent with the study by Farouk and colleagues, (2022) which reported a significant increase in the total score of nurses’ practices following an intervention. Their research found that the proportion of nurses with competent practices increased markedly post-intervention, demonstrating the effectiveness of educational programs in improving practical skills. Such alignment with previous research reinforces the validity and generalizability of the current study’s findings [16]. Similarly, the study by Shekoohiyan et al., (2022) aligns with these results. Their research, conducted during the COVID-19 pandemic, assessed the knowledge, attitude, and practice of healthcare staff regarding biomedical waste management in Fasa educational hospitals. They reported a significant overall improvement in waste management practices across various healthcare facilities, further supporting the effectiveness of targeted interventions. The unique context of their study, occurring during a global health crisis, highlights the adaptability and broad applicability of such educational strategies across diverse healthcare settings [20].

The findings also align with those of Hosny et al. (2018) and Kaur et al. (2023), emphasizing the critical role of training and educational programs in enhancing healthcare workers’ practices and competence in biomedical waste management. Hosny et al. observed substantial improvements in practice categories post-training, while Kaur et al. demonstrated the effectiveness of targeted interventions in improving knowledge, attitudes, and practices among staff nurses and students, reinforcing the importance of educational efforts in this domain [17, 21].

The correlation between the studied nurses’ total knowledge and practices regarding biomedical waste management revealed a positive significant correlation where (*r* = 0.62) at (*P* < 0.001). These findings suggest that as nurses’ knowledge about biomedical waste management improved, their practical implementation of waste management protocols also showed considerable enhancement.

The study by Shivashankarappa et al. (2024) supports these results, showing a significant difference between pre- and post-interventional knowledge and practices regarding biomedical waste management. The consistency of these findings across different studies emphasizes the importance of continuous training and education in improving biomedical waste management practices among healthcare workers. These studies collectively reinforce the idea that targeted interventions can effectively bridge the knowledge-practice gap and promote sustainable improvements in healthcare practices [22].

However, these findings contrast with those of El-Naggar et al. (2017), who reported a negative correlation between knowledge and practices in their study on medical waste management training in Zagazig University hospitals [23]. Their study suggests that, despite increased knowledge, practical implementation of waste management practices was not significantly enhanced, possibly due to other factors such as lack of proper resources, inadequate reinforcement of learned concepts, or insufficient organizational support. This contrast highlights the need for a more comprehensive approach that includes not only education but also resource provision, continuous monitoring, and institutional support to ensure that improvements in knowledge translate into actual practice.

## Conclusion

This study highlights the critical role of educational interventions in improving nurses’ knowledge and practices regarding biomedical waste management. The findings demonstrate that the intervention led to significant improvements in both knowledge and competent practices, emphasizing the effectiveness of structured training programs. Beyond individual healthcare institutions, integrating biomedical waste management training into nursing education policies at national and institutional levels can help sustain these improvements. Embedding such content into nursing curricula and continuing education programs ensures long-term competency, reinforcing safe and sustainable waste management practices in diverse healthcare settings.

### Implications

The study underscores the need for structured educational interventions, supported by clear policies and ongoing monitoring, to enhance nurses’ biomedical waste management practices. Beyond hospital settings, integrating waste management training into nursing curricula at national and institutional levels can ensure long-term competency and sustainability. Further research should explore factors influencing knowledge translation into practice, while tailored interventions can provide a globally applicable solution.

### Limitations of the study

While this study demonstrates the effectiveness of an educational program in enhancing nursing competence in biomedical waste management, there are some limitations. The study was conducted in a single hospital with a sample of 75 nurses, which may limit the broader applicability of the findings. Additionally, the use of self-reported data may introduce some bias in the assessment of practices. The lack of a control group and the short-term follow-up assessment also means that the long-term sustainability of the intervention’s impact could not be fully determined. Despite these factors, the findings offer valuable insights into the role of structured educational programs in improving nursing practices in biomedical waste management.

## Data Availability

The corresponding author can provide the datasets used and/or analyzed for this study upon reasonable request.

## Abbreviations

BMW: Biomedical waste
REC: Research ethics committee
HCWs: Healthcare workers
